# Supplementary material for: Transient photocurrents in a subthreshold evidence accumulator accelerate perceptual decisions
Source: Nat Commun. 2023 May 13;14:2770. doi: 10.1038/s41467-023-38487-5 (PMC10182991; doi:10.1038/s41467-023-38487-5)
Supplement: Supplementary file 1 — Supplementary Information [file 41467_2023_38487_MOESM1_ESM.pdf]

Supplementary Information

Transient Photocurrents in a Subthreshold Evidence Accumulator Accelerate Perceptual Decisions

Timothy L. H. Wong[1], Clifford B. Talbot[1], and Gero Miesenböck[1*]

[1] Centre for Neural Circuits and Behavior, University of Oxford, Tinsley Building, Mansfield Road, Oxford OX1 3SR, United Kingdom

These authors contributed equally: Timothy L. H. Wong, Clifford B. Talbot

[*] Correspondence: gero.miesenboeck@cncb.ox.ac.uk

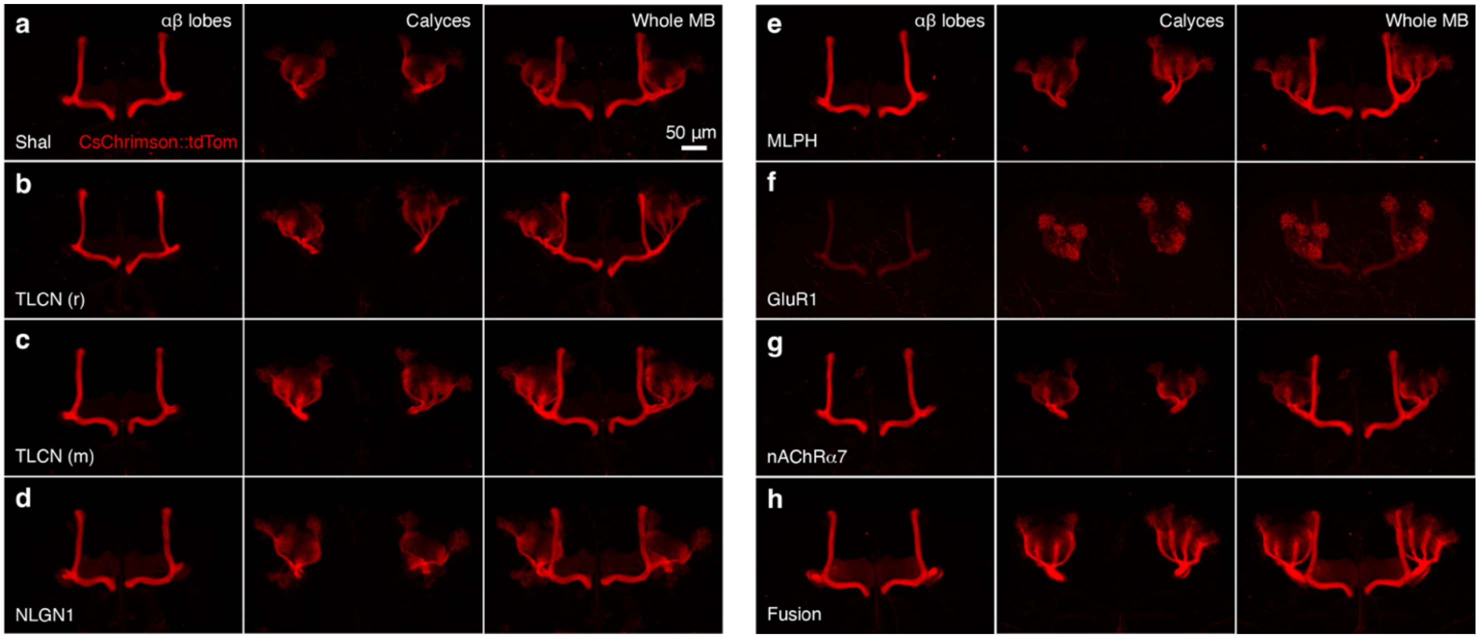

Supplementary Fig. 1 | Dendritic targeting of CsChrimson.
a–**h**, Native tdTomato fluorescence in the αβ lobes (left), calyces (center), and whole mushroom bodies (right) of flies expressing *NP6024-GAL4*-driven *UAS* transgenes encoding CsChrimson::tdTomato fusion proteins that carried localization motifs from Shal (**a**), rabbit (r) or mouse (m) telencephalin (TLCN; **b** and **c**), neuroligin 1 (NLGN1; **d**), melanophilin (MLPH; **e**), the GluR1 subunit of the AMPA receptor (GluR1; **f**), the α7 subunit of the nicotinic acetylcholine receptor (nAChRα7; **g**), and a fusion of TLCN and K_v2.1 (**h**). The images show representative examples of 6 (**a**), 5 (**b**), 4 (**c**), 6 (**d**), 6 (**e**), 5 (**f**), 6 (**g**), and 6 brains (**h**).

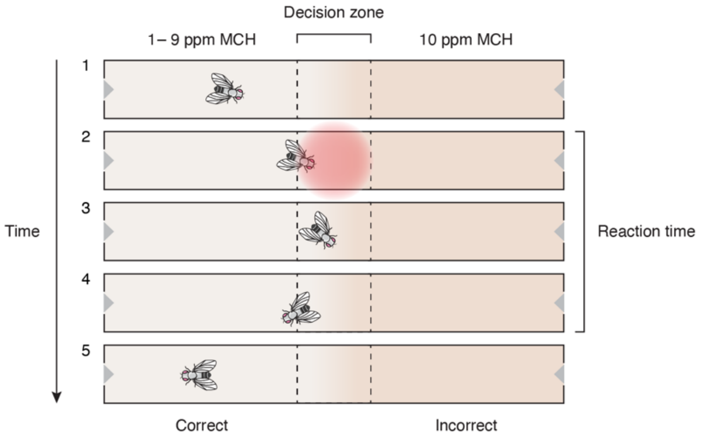

Supplementary Fig. 2 | Measurement of reaction times and choice accuracies.
Two odor streams converge in a 7 mm wide decision zone at the center of a narrow chamber. Flies are trained to avoid 10 ppm of 4-methylcyclohexanol (MCH) and must distinguish the reinforced MCH concentration from a lower intensity of the same odor (1–9 ppm); the concentration ratio during testing determines the difficulty of discrimination. The temporal sequence shown in frames 1–5 illustrates a single decision schematically. Entry of the fly into the decision zone (frame 2) starts the reaction time clock and, under photostimulation conditions, triggers a single 25-ms pulse of red light at 0.01 or 0.20 mW mm^{-2}; the reaction time clock stops when the fly leaves the decision zone (frame 4). Exits into the lower MCH concentration are scored as correct choices (frame 5); exits into the aversively reinforced concentration of 10 ppm MCH constitute errors.

Supplementary Table 1 | Sample sizes for behavioral measurements in Fig. 2.

Optical power	Odor contrast	Genotype: *NP6024-GAL4 > UAS–CsChrimson::GluR1*		Genotype: *NP6024-GAL4 > UAS–CsChrimson::NLGN1*	
		Flies (*m*)	Decisions (*n*)	Flies (*m*)	Decisions (*n*)
0 mW mm^{-2}	10 : 100	55	514	38	382
	40 : 100	79	794	57	651
	50 : 100	53	601	62	598
	70 : 100	122	1084	61	536
	90 : 100	114	915	60	495
	100 : 90	117	817	63	484
	100 : 70	115	957	59	546
	100 : 50	51	577	57	603
	100 : 40	63	677	56	647
	100 : 10	53	567	37	376
0.01 mW mm^{-2}	10 : 100	30	350	49	523
	40 : 100	49	530	44	490
	50 : 100	50	509	42	451
	70 : 100	57	521	78	735
	90 : 100	49	358	46	388
	100 : 90	95	748	44	392
	100 : 70	47	479	52	542
	100 : 50	46	527	54	556
	100 : 40	55	670	62	707
	100 : 10	29	356	42	425
0.20 mW mm^{-2}	10 : 100	38	437	31	189
	40 : 100	43	498	52	327
	50 : 100	41	382	51	349
	70 : 100	58	530	70	365
	90 : 100	41	326	67	378
	100 : 90	81	711	62	285
	100 : 70	60	631	69	393
	100 : 50	39	366	56	377
	100 : 40	36	348	53	384
	100 : 10	44	449	47	266

Supplementary Table 2 | Sample sizes for behavioral measurements in Fig. 3.

Optical power	Odor contrast	Genotype			
		VT030604-GAL4 > UAS–CsChrimson::GluR1		*NP6024-GAL4*	
		Flies (*m*)	Decisions (*n*)	Flies (*m*)	Decisions (*n*)
0 mW mm^{-2}	10 : 100	70	517	43	325
	40 : 100	85	600	43	349
	50 : 100	46	306	37	332
	70 : 100	100	531	47	403
	90 : 100	96	463	30	202
	100 : 90	94	432	48	370
	100 : 70	84	406	43	346
	100 : 50	49	296	42	323
	100 : 40	91	600	39	359
	100 : 10	56	421	38	295
0.01 mW mm^{-2}	10 : 100	37	329	22	151
	40 : 100	55	416	42	356
	50 : 100	33	225	30	269
	70 : 100	72	427	36	285
	90 : 100	65	324	54	428
	100 : 90	55	285	37	285
	100 : 70	68	384	29	306
	100 : 50	36	197	31	294
	100 : 40	60	444	43	402
	100 : 10	35	282	26	193

Supplementary Table 3 | Primer sequences.

PCR

Motif	Forward primer (5' - 3')	Reverse primer (5' - 3')
Shal	ATAAGAATGCGGCCGCATGAGCAGACTGGTCGCCGCT	TCCAATGCATTGGTTATTATTACGTTGTCTTCTCCAGACATCGCAGCAAATGATGGTGCTGCAGCTCGAAGCCGCCGGAGCCGCCGGATTTATACAGTTCGTCCATACC
TLCN (r)	ATAAGAATGCGGCCGCATGAGCAGACTGGTCGCCGCT	TCCAATGCATTGGTTATTATTAGGCGGAGGTCAGCTGGATGGCGAACACCTCGCCGCCGGCGGGGGACTCGGCGCCGCCGGAGCCGCCGGATTTATACAGTTCGTCCATACC
TLCN (m)	ATAAGAATGCGGCCGCATGAGCAGACTGGTCGCCGCT	TCCAATGCATTGGTTATTATTAGGAGGAGGTCAGCTGGATGGCGAACACCTCGCCGTCGGCGGGGGACTCGGCGCCGCCGGAGCCGCCGGATTTATACAGTTCGTCCATACC

GeneArt

Motif	Construct sequence
NLGN1	CTCGAGGTGGTGCTGCGCACCGCCTGCCCCCCCGACTACACCCTGGCCATGCGCCGCTCCCCCGACGACGTGCCCCTGATGACCCCCAACACCATCACCATGTAATAATAAGGTACC
MLPH	CTCGAGCGCGACCAGCCCCTGAACTCCAAGAAGAAGAAGCGCCTGCTGTCCTTCCGCGACGTGGACTTCGAGGAGGACTCCGACTAATAATAAGGTACC
GluR1	CTCGAGGAGTTCTGCTACAAGTCCCGCTCCGAGTCCAAGCGCATGAAGGGCTTCTGCCTGATCCCCCAGCAGTCCATCAACGAGGCCATCCGCACCTCCACCCTGCCCCGCAACTCCGGCGCCTAATAATAAGGTACC
nAChRα7	CTCGAGGGCGAGGACAAGGTGCGCCCCGCCTGCCAGCACAAGCCCCGCCGCTGCTCCCTGGCCTCCGTGGAGCTGTCCGCCGGCGCCGGCCCCCCCACCTCCAACGGCAACCTGCTGTACATCGGCTTCCGCGGCCTGGAGGGCATGCACTAATAATAAGGTACC
Fusion	CTCGAGCAGTCCCAGCCCATCCTGAACACCAAGGAGATGGCCCCCCAGTCCAAGCCCCCCGAGGAGCTGGAGATGTCCTCCATGCCCTCCCCCGTGGCCCCCCTGCCCGCCCGCACCGAGGGCGTGATCGACATGCGCTCCATGTCCTCCATCGACTCCTTCATCTCCTGCGCCACCGACTTCCCCGAGGCCACCCGCTTCGGCTCCGGCTCCGGCTCCGGCTCCGGCTCCGCCGAGTCCCCCGCCGACGGCGAGGTGTTCGCCATCCAGCTGACCTCCTCC TAATAATAAGGTACC

Fusion PCR

Construct	Forward primer (5' - 3')	Reverse primer (5' - 3')
CsChrimson::NLGN1	ATAAGAATGCGGCCGCATGAGCAGACTGGTCGCCGCT	GTCGGGGGGGCAGGCGGTGCGCAGCACCACGCCGCCGGAGCCGCCGATTTATACAGTTCGTCCATACC
CsChrimson::NLGN1	TTTCTGTATGGTATGGACGAACTGTATAAATCCGGCGGCTCCGGCGGCGTGGTGCTGCGCACCGCCTGC	TCCAATGCATTGGGGTACCTTATTATTACATGGTGATGGTGTT
CsChrimson::MLPH	ATAAGAATGCGGCCGCATGAGCAGACTGGTCGCCGCT	CTTCTTCTTGGAGTTCAGGGGCTGGTCGCGGCCGCCGGAGCCGCCGGATTTATACAGTTCGTCCATACC
CsChrimson::MLPH	TTTCTGTATGGTATGGACGAACTGTATAAATCCGGCGGCTCCGGCGGCCGCGACCAGCCCCTGAACTCC	TCCAATGCATTGGGGTACCTTATTATTAGTCGGAGTCCTCCTC
CsChrimson::GluR1	ATAAGAATGCGGCCGCATGAGCAGACTGGTCGCCGCT	GGACTCGGAGCGGGACTTGTAGCAGAACTCGCCGCCGGAGCCGCCGGATTTATACAGTTCGTCCATACC
CsChrimson::GluR1	TTTCTGTATGGTATGGACGAACTGTATAAATCCGGCGGCTCCGGCGGCGAGTTCTGCTACAAGTCCCGC	TCCAATGCATTGGGGTACCTTATTATTAGGCGCCGGAGTTGCG
CsChrimson::nAChRα7	ATAAGAATGCGGCCGCATGAGCAGACTGGTCGCCGCT	CTGGCAGGCGGGGCGCACCTTGTCCTCGCCGCCGCCGGAGCCGCCGGATTTATACAGTTCGTCCATACC
CsChrimson::nAChRα7	TTTCTGTATGGTATGGACGAACTGTATAAATCCGGCGGCTCCGGCGGCGGCGAGGACAAGGTGCGCCCC	TCCAATGCATTGGGGTACCTTATTATTAGTGCATGCCCTCCAG
CsChrimson::fusion	ATAAGAATGCGGCCGCATGAGCAGACTGGTCGCCGCT	CTCCTTGGTGTTCAGGATGGGCTGGGACTGGCCGCCGGAGCCGCCGGATTTATACAGTTCGTCCATACC
CsChrimson::fusion	TTTCTGTATGGTATGGACGAACTGTATAAATCCGGCGGCTCCGGCGGCCAGTCCCAGCCCATCCTGAAC	TCCAATGCATTGGGGTACCTTATTATTAGGAGGAGGTCAGCTG
