# Supplementary material for: Transient photocurrents in a subthreshold evidence accumulator accelerate perceptual decisions
Source: Nat Commun. 2023 May 13;14:2770. doi: 10.1038/s41467-023-38487-5 (PMC10182991; doi:10.1038/s41467-023-38487-5)
Supplement: Supplementary file 3 — Reporting Summary [file 41467_2023_38487_MOESM3_ESM.pdf]

nature portfolio

Corresponding author(s): Gero Miesenböck

Last updated by author(s): Apr 21, 2023

Reporting Summary

Nature Portfolio wishes to improve the reproducibility of the work that we publish. This form provides structure for consistency and transparency in reporting. For further information on Nature Portfolio policies, see our Editorial Policies and the Editorial Policy Checklist.

Statistics

For all statistical analyses, confirm that the following items are present in the figure legend, table legend, main text, or Methods section.

n/a	Confirmed	
☐	☒	The exact sample size (*n*) for each experimental group/condition, given as a discrete number and unit of measurement
☐	☒	A statement on whether measurements were taken from distinct samples or whether the same sample was measured repeatedly
☐	☒	The statistical test(s) used AND whether they are one- or two-sided<br>*Only common tests should be described solely by name; describe more complex techniques in the Methods section.*
☒	☐	A description of all covariates tested
☐	☒	A description of any assumptions or corrections, such as tests of normality and adjustment for multiple comparisons
☐	☒	A full description of the statistical parameters including central tendency (e.g. means) or other basic estimates (e.g. regression coefficient) AND variation (e.g. standard deviation) or associated estimates of uncertainty (e.g. confidence intervals)
☐	☒	For null hypothesis testing, the test statistic (e.g. *F*, *t*, *r*) with confidence intervals, effect sizes, degrees of freedom and *P* value noted<br>*Give P values as exact values whenever suitable.*
☐	☒	For Bayesian analysis, information on the choice of priors and Markov chain Monte Carlo settings
☒	☐	For hierarchical and complex designs, identification of the appropriate level for tests and full reporting of outcomes
☒	☐	Estimates of effect sizes (e.g. Cohen's *d*, Pearson's *r*), indicating how they were calculated

Our web collection on statistics for biologists contains articles on many of the points above.

Software and code

Policy information about availability of computer code

Data collection	For the collection of behavioral data, Raspberry Pis were running OS Raspbian 10 and custom software written in Python 3.6.1 with modules installed using Berryconda: scipy 1.0.0, pyserial 3.4, pillow 3.4.1 and picamera 1.13. Arduino code was written in C using the Arduino IDE version 1.8.10. The data acquisition PC was running Windows 7, with custom software in LabVIEW 2012.<br>Electrophysiology data were acquired using pCLAMP 10.
Data analysis	Python 3.7.9, MATLAB 2019b, Igor Pro 8, Prism 9.<br>Custom code is available at<br>https://doi.org/10.5281/zenodo.7853204

For manuscripts utilizing custom algorithms or software that are central to the research but not yet described in published literature, software must be made available to editors and reviewers. We strongly encourage code deposition in a community repository (e.g. GitHub). See the Nature Portfolio guidelines for submitting code & software for further information.

Data

Policy information about availability of data

All manuscripts must include a data availability statement. This statement should provide the following information, where applicable:

- Accession codes, unique identifiers, or web links for publicly available datasets
- A description of any restrictions on data availability
- For clinical datasets or third party data, please ensure that the statement adheres to our policy

Source data are provided with this paper. Behavioral data are also available at https://doi.org/10.5281/zenodo.7853204

Human research participants

Policy information about studies involving human research participants and Sex and Gender in Research.

Reporting on sex and gender	N/A
Population characteristics	N/A
Recruitment	N/A
Ethics oversight	N/A

Note that full information on the approval of the study protocol must also be provided in the manuscript.

Field-specific reporting

Please select the one below that is the best fit for your research. If you are not sure, read the appropriate sections before making your selection.

☒ Life sciences ☐ Behavioural & social sciences ☐ Ecological, evolutionary & environmental sciences

For a reference copy of the document with all sections, see nature.com/documents/nr-reporting-summary-flat.pdf

Life sciences study design

All studies must disclose on these points even when the disclosure is negative.

Sample size	No statistical methods were used to predetermine sample sizes, which were based on prior studies (DasGupta et al., Science 2014; Groschner et al., Cell 2018; Vrontou et al., Curr. Biol. 2021).
Data exclusions	Immobile flies (<2 runs into the decision zone) as well as flies that excessively groomed in the decision zone were excluded.
Replication	Because of the large sample sizes for each difficulty level, light condition, and genotype, behavioral experiments were not replicated.
Randomization	For each fly, the task difficulty and photostimulation conditions were chosen at random.
Blinding	The investigators were not blinded to group allocation. Blinding was unnecessary because the collection and analysis of reaction time and choice accuracy data was fully automated, with no scope for human intervention.

Reporting for specific materials, systems and methods

We require information from authors about some types of materials, experimental systems and methods used in many studies. Here, indicate whether each material, system or method listed is relevant to your study. If you are not sure if a list item applies to your research, read the appropriate section before selecting a response.

Materials & experimental systems

n/a	Involved in the study
☒	☐ Antibodies
☒	☐ Eukaryotic cell lines
☒	☐ Palaeontology and archaeology
☐	☒ Animals and other organisms
☒	☐ Clinical data
☒	☐ Dual use research of concern

Methods

n/a	Involved in the study
☒	☐ ChIP-seq
☒	☐ Flow cytometry
☒	☐ MRI-based neuroimaging

Animals and other research organisms

Policy information about studies involving animals; ARRIVE guidelines recommended for reporting animal research, and Sex and Gender in Research

Laboratory animals	Drosophila melanogaster (transgenic strains described in Methods) males aged 1-2 and 7-10 days were used for electrophysiology and behavioral experiments, respectively.
Wild animals	No wild animals were used in this study.
Reporting on sex	Findings are expected to generalize to both sexes. Only male flies were used in behavioral experiments.
Field-collected samples	No field-collected samples were used in this study.
Ethics oversight	No ethical approval or guidance was required for studies on Drosophila.

Note that full information on the approval of the study protocol must also be provided in the manuscript.
